# Healthcare resources, organisational support and practice in asthma in six public health clinics in Malaysia

**DOI:** 10.1038/s41533-023-00337-8

**Published:** 2023-03-27

**Authors:** Norita Hussein, Rizawati Ramli, Su May Liew, Nik Sherina Hanafi, Ping Yein Lee, Ai Theng Cheong, Shariff-Ghazali Sazlina, Azainorsuzila Mohd Ahad, Jaiyogesh Patel, Jürgen Schwarze, Hilary Pinnock, Ee Ming Khoo

**Affiliations:** 1 Department of Primary Care Medicine, Faculty of Medicine, Universiti Malaya, Kuala Lumpur, Malaysia; 2 UMeHealth Unit, Faculty of Medicine, Universiti Malaya, Kuala Lumpur, Malaysia; 3 Department of Family Medicine, Faculty of Medicine and Health Sciences, Universiti Putra Malaysia, Serdang, Malaysia; 4 Port Dickson Health Clinic, Ministry of Health, Port Dickson, Malaysia; 5 NIHR Global Health Research Unit on Respiratory Health (RESPIRE), Usher Institute, Usher Institute, The University of Edinburgh, Edinburgh, UK; 6 Child Life and Health, Centre for Inflammation Research, The University of Edinburgh, Edinburgh, UK

**Keywords:** Health services, Asthma

## Abstract

Asthma, a common chronic respiratory illness is mostly managed in primary care. We aimed to determine healthcare resources, organisational support, and doctors’ practice in managing asthma in a Malaysian primary care setting. A total of six public health clinics participated. We found four clinics had dedicated asthma services. There was only one clinic which had a tracing defaulter system. Long-term controller medications were available in all clinics, but not adequately provided. Resources, educational materials, and equipment for asthma management were present, though restricted in number and not placed in main locations of the clinic. To diagnose asthma, most doctors used clinical judgement and peak flow metre measurements with reversibility test. Although spirometry is recommended to diagnose asthma, it was less practiced, being inaccessible and unskilled in using as the main reasons. Most doctors reported providing asthma self-management; asthma action plan, but for only half of the patients that they encountered. In conclusion, there is still room for improvement in the provision of clinic resources and support for asthma care. Utilising peak flow metre measurement and reversibility test suggest practical alternative in low resource for spirometry. Reinforcing education on asthma action plan is vital to ensure optimal asthma care.

## Introduction

Asthma is the most common chronic respiratory illness affecting an estimated 262 million people worldwide^1^. In Malaysia, the prevalence is estimated between 8.9 and 13.0% in children^2,3^ and 6.3% in adults^4^. Despite evidence-based asthma management recommendations and treatments, asthma control is still suboptimal. A recent local study showed only 37% had well-controlled asthma, 36% were partly controlled and 27% uncontrolled^5^. Primary care is ideally placed to diagnose, manage and provide continuous care to patients with asthma^6,7^. Important factors that have been reported to facilitate improvement in primary care management of asthma include availability of good organisational support and access to resources within the practice, as well as having a dedicated asthma team (doctors, pharmacists, nurses, allied health professional trained in asthma)^8^. However, it is not clear to what degree these factors are being provided to facilitate asthma management in our Malaysian primary care setting. We therefore aimed to determine current resources available, organisational support and doctors’ provision of asthma care in our public health clinics. The findings will help inform strategies to improve the delivery of asthma care in our setting.

## Methods

### Study design, setting and participants

This is a cross-sectional survey conducted from December 2019 to January 2020 in six public health clinics in Klang District, Malaysia. Malaysia has a dual-sector healthcare system: public and private sectors^9^. The public health clinics are government-funded with heavily subsidised in which patient pays MYR1 (USD0.24) per clinic visit that covers the cost of consultation, investigations, and medications while government employees, pensioners, school-going children and people aged 60 years and above receive free health services^10^. On the other hand, the private health clinics operate on a fee for service^9^. The Klang District has mainly a low-middle socioeconomic class population and chronic disease care is mainly provided from public health clinics^11^. Every public health clinic is led by Family Medicine Specialists, and are staffed by various healthcare providers: doctors, nurses, pharmacists, medical assistants and lab technicians. There were nine Family Medicine Specialists and 107 doctors at the time of study, all of whom were invited to participate. Written informed consent was obtained from every participant prior to carrying out the study.

### Sampling method

The six clinics were purposively sampled to define characteristics that address the settings of low-middle income demographic. The characteristics of these clinics include government-subsidised and catered for a population which, majority were from low-middle income socio-economic groups. The size of population served by each clinic ranged from 82,000 to 156,672 people. Klang District is one of the most densely populated districts in Malaysia^12^.

### Data collection

We used a self-administered questionnaire to collect the data. The questionnaire was developed based on a literature review, training module on asthma for healthcare providers at the primary care level in Malaysia^13^ and the views of an expert panel comprising a team of Family Medicine Specialists, respiratory physicians, and academicians in primary care medicine. Component 1 of the questionnaire includes questions on healthcare resources, clinic organisation as well as asthma services and treatment, which includes the availability of emergency care, training on asthma for healthcare providers, frequency of asthma clinical audits and availability of asthma medications. The number of resources and equipment available in designated areas in the clinic related to asthma was also collected. The questionnaire for Component 2 asked about the doctors’ current practice on asthma management including number of years in service, number of patients with asthma seen in a month, tools used to diagnose asthma, use of spirometry, peak flow metre and asthma guidelines. The questionnaire was given to six Family Medicine Specialists, two respiratory physicians and five doctors to ensure its face and content validity and underwent a series of revisions where items were evaluated for clarity, easy comprehension, and formatting. The layout of the clinic areas was checked by the researchers prior to this study to better understand the availability of current resources and organisational support. This includes the layout of consultation rooms, treatment rooms (for nebulisation, immunisation, wound dressing), emergency room, pharmacy, laboratory, meeting room and room for continuous medical education (CME).

### Data analysis

Statistical analysis was done using SPSS version 25.0. Descriptive statistics were used, where data were described using frequencies and percentages for categorical variables or means with standard deviation (SD) for continuous variables.

### Ethical approval

We obtained ethical approval from the National Medical Research Ethics Committee, Ministry of Health, Malaysia (NMRR-18-2707-42719) and sponsor approval from the Academic and Clinical Central Office for Research and Development, University of Edinburgh United Kingdom (AC19040).

### Reporting summary

Further information on research design is available in the Nature Portfolio Reporting Summary linked to this article.

## Results

### Component 1: healthcare resources and organisational support in asthma management

All data collected for Component 1 were captured objectively from patient registries, existing available clinic infrastructure and resources; and were reported by the Family Medicine Specialists from the six public health clinics. Table 1 summarises the provision and availability of healthcare resources and organisational support in asthma management. The average number of patients with asthma registered in each clinic ranged from 100 to 311 for adults and 30 to 100 for children and adolescents (less than 18 years old). However, two clinics reported they did not have registries for children or adolescents with asthma at the time of data collection. Most practices had dedicated asthma clinics which operated once a week or once a fortnight. These dedicated asthma clinics were run by trained healthcare personnel. An asthma appointment system was available in five clinics; however, only one had a system for tracing or recalling patients who defaulted follow-up. To enhance patients’ adherence to treatment, four clinics had initiated an Asthma Medication Therapy Adherence Clinic (MTAC) run mainly by pharmacists. Oral corticosteroids for acute attacks and long-term controller medications, inhaled corticosteroids (ICS), and combination of inhaled corticosteroids and long-acting beta-agonist (ICS/LABA) were available but inadequate in quantities to meet the demand in the clinics. Leukotriene receptor antagonists (LTRA) were not available in any of the clinics. All clinics have a Critical Care Patient Escort and Retrieval Team (CPERT) system, which had been implemented to facilitate immediate referral of emergency cases to the tertiary centre when this system is activated. CPERT is a system unique to the district of Klang, Malaysia.

**Table 1:** Healthcare resources and organisational support in asthma management in the six public health clinics (*N* = 6).

*1. Organisation of asthma services*	
Total number of patients in each clinic (*N*)	(Adult/children & adolescents)
Clinic 1	240/not available
Clinic 2	288/not available
Clinic 3	300/100
Clinic 4	237/50
Clinic 5	100/30
Clinic 6	311/32
Availability of dedicated asthma clinic	4 clinics
Frequency of asthma clinic being held	Once a week or once every 2 weeks
Availability of asthma team	5 clinics
Comprises of Family Medicine Specialists (FMS), doctors, nurses, pharmacists and medical assistants (MA)	For each asthma team: -All 5 clinics have *FMS (*n* = 1 or 2), doctors (*n* = 2) and nurses (*n* = 2). -4 clinics have pharmacists (*n* = 1) -3 clinics have medical assistants (*n* = 1)
Received formal asthma training	All healthcare personnel in the asthma team
Availability of asthma appointment system	5 clinics
Availability of defaulter tracing or recall system	1 clinic
Availability of Medication Therapy Adherence Clinic (MTAC)^#^ programme for asthma	4 clinics
Number of patients with asthma registered in past year, mean (range)	35 (1–110)
Proportion of patients with asthma (%) provided with asthma record book, mean (range)	57 (40–100)
Healthcare personnel who carried out assessment of asthma control	All doctors in all clinics Nurses and pharmacists in 3 clinics
*2. Emergency care*	
Availability of emergency medications	
Hydrocortisone, Prednisolone, Adrenaline, Salbutamol nebulised, Salbutamol/ipratropium nebulised	All clinics
Availability of ambulance services	All clinics
Availability of Critical Care Patient Escort and Retrieval Team (CPERT)^§^ system	All clinics
*3. Availability of long-term asthma treatment*	
Availability of asthma medications	
1. Inhaled corticosteroids (ICS)	All clinics
Supply adequate?	Yes
2. Combination Long-Acting Beta-Agonist (LABA)/ Inhaled Corticosteroid (ICS) (Seretide and Symbicort)	All clinics
Supply adequate?	No
3. Theophylline	All clinics
Supply adequate?	Yes
4. Leukotriene receptor antagonist (LTRA)	Not available
*4. Asthma training and audit*	
Availability of asthma training (course/workshop)	All clinics
Frequency/year (range)	1–3 times/year
Availability of clinic audit for asthma care	All clinics
Frequency/year (range)	1–12 times/year
Availability of national-level Quality Assurance^+^ for asthma	All clinics
Frequency/year (range)	1 time/year

^#^MTAC Medication Therapy Adherence Clinic—introduced in Malaysia in 2004 as a component of ambulatory care, with the aim to improve patient adherence to medication.

^§^CPERT Critical Care Patient Escort and Retrieval Team system to facilitate immediate referral of emergency cases to tertiary centre when this system is activated.

^+^Quality Assurance are measures taken by the specific practice to ensure the quality of care for patients with asthma are standardised throughout the practice, especially when involving many different doctors treating the patients.

*Three clinics have 2 FMSs and another two clinics have 1 FMS each.

**Fig. 1:**
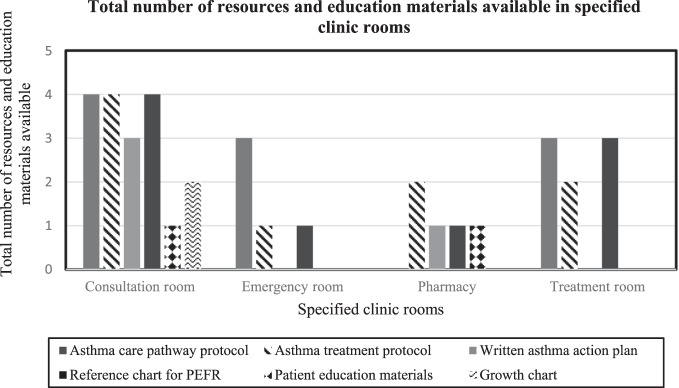
The total number of resources and education materials available in specified clinic rooms (consultation room, emergency room, pharmacy and treatment room) in all clinics. PEFR peak expiratory flow rate.

**Fig. 2:**
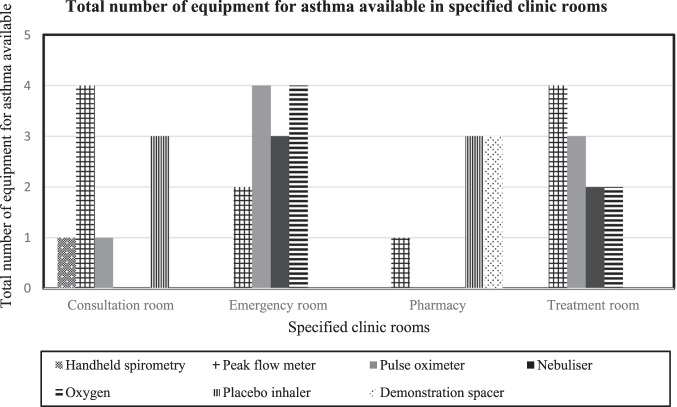
The total number of equipment for asthma available in specified clinic rooms (consultation room, emergency room, pharmacy and treatment room) in all clinics.

All six clinics have about similar layout. Each clinic has between 10 and 20 consultation rooms, one emergency room, one pharmacy and one consultation room for pharmacist, and one treatment room. Tables 2 and 3 summarise the total number of clinics with resources, educational materials and equipment for asthma and its availability in specified clinic rooms. Figures 1 and 2 demonstrate the availability of resources, education materials and equipment in the specified clinic rooms (consultation rooms, emergency rooms, pharmacies and treatment rooms). All clinics reported to have asthma care pathway and asthma treatment protocols, written asthma action plans, peak expiratory flow rate (PEFR) reference chart, children&rsqio;s growth chart, peak flow metres, pulse oximeters, nebulisers, oxygen supply and placebo inhalers. Although the resources, educational materials and equipment were provided in all clinics, the availability and placement of them varied. The clinics often had the materials and equipment they need, but not necessarily in the right places. Spacers were available in four clinics, and one clinic had handheld spirometry. Only three clinics had patient education leaflets for asthma.

**Table 2:** Total number of clinics with resources and educational materials for asthma and its availability in specified clinic rooms.

Resources and education materials	Asthma care pathway protocol	Asthma treatment protocol	Written AAP	PEFR reference chart	*Patient education leaflet	Growth chart for children
Availability in clinics (*N* = 6)	All clinics	All clinics	All clinics	All clinics	3 clinics	All clinics
Consultation room	√	√	√	√	√	√
Emergency room	√	√		√		
Pharmacy		√	√	√	√	
Treatment room	√	√		√		

*AAP* asthma action plan, *PEFR* peak expiratory flow rate.

*Patient education leaflet on asthma can be accessed from the Malaysia Ministry of Health website:

http://www.myhealth.gov.my/en/asthma-2-2/.

**Table 3:** Total number of clinics with equipment for asthma and its availability in specified clinic rooms.

Equipment	Handheld spirometry	Peak flow metre	Pulse oximeter	Nebuliser	Oxygen	Placebo inhaler	Spacer
Availability in clinics *N* = 6	1 clinic	All clinics	All clinics	All clinics	All clinics	All clinics	4 clinics
Consultation room	√	√	√			√	
Emergency room		√	√	√	√		
Pharmacy		√				√	√
Treatment room		√	√	√	√

### Component 2: doctors’ practice on asthma

A total of 107 doctors were invited to participate, however only 100 completed questionnaires were returned (94% response rate). Table 4 summarises the doctors’ profile and how long they had been managing asthma. The average number of patients with asthma seen in a month was 19, most of whom they diagnosed by clinical assessment and peak flow metre measurements; with 65% of the doctors experienced carrying out a reversibility test with a peak flow metre. Only 18% used spirometry to diagnose asthma. The main reason for poor usage of spirometry was a lack of accessibility and familiarity. Peak flow rate was mainly assessed and documented during routine follow-up, and only about two-thirds of the doctors carried out peak flow assessment during unscheduled visits (potentially for an attack). History and examination (84%) and GINA assessment of asthma control (80%) were the main strategies for assessing asthma control. Most reported providing asthma action plans during consultations, though not to all the patients that they reviewed.

**Table 4:** Profile of doctors and their asthma practice (*N* = 100).

Profile	*n* (%)
Number of years working as a doctor, mean (SD)	10.1 (4.2)
Number of years working in primary care clinic, mean (SD)	6.0 (4.1)
Number of patients with asthma seen in a month, mean (range)	19 (1–80)
Methods used to diagnose asthma	
Clinical (History and physical examination)	96 (96.0)
Peak flow metre	84 (84.0)
Spirometry	18 (18.0)
Chest X-ray	5 (5.0)
Other (family history, oxygen saturation levels)	2 (2.0)
Number of doctors who had used spirometry to diagnose asthma:	18 (18.0)
If NOT, why	
Lack of accessibility	80 (80.0)
Lack of familiarity	20 (20.0)
Do not know how to interpret	10 (10.0)
Costly	4 (4.0)
Number of doctors who have carried out peak flow metre and reversibility test to diagnose asthma	65 (65.0)
Commonly peak flow expiratory rate (PEFR) measurement was carried out during	
Follow-up	96 (96.0)
Acute exacerbation	60 (60.0)
Walk-in	57 (57.0)
Number of doctors who reported prescribing asthma action plans	91 (91.0)
Mean (SD)	5.71 (3.46)
Main tool used for the assessment of asthma control	
History and examination	84 (84.0)
Global Initiative for Asthma (GINA) Guidelines	80 (80.0)
Peak Expiratory Flow Rate (PEFR)	77 (77.0)
Malaysian Clinical Practice Guidelines on Asthma	46 (46.0)
Asthma Control Test (ACT)	35 (35.0)
Asthma Control Questionnaires (ACQ)	11 (11.0)
Main asthma management guidelines used	
Global Initiative for Asthma (GINA) Guidelines	96 (96.0)
Malaysian Clinical Practice Guidelines on Asthma	63 (63.0)
National Institute for Health and Care Excellence (NICE) Guideline	8 (8.0)

## Discussion

This study indicates there is room for improvement in the existing healthcare resources, organisational support and practices on asthma care. Four of the six clinics had dedicated asthma services run by teams of trained healthcare personnel. Five clinics had appointment systems for registered patients with asthma, however, only one clinic had a defaulter tracing system. All clinics had satisfactory provision of charts, tools, and equipment (e.g., peak flow metre, pulse oximeter, nebuliser machines) appropriate for asthma management, however, the availability in specified clinic rooms varied and appeared not to be readily available in important areas for use when needed during consultation or emergency care. Long-term controller medications for asthma for example ICS and combination ICS/LABA were available, though the latter were not adequately provided. To enhance compliance, four clinics had implemented a Medication Therapy Adherence Clinic (MTAC) programme for asthma run by pharmacists. Clinical assessment and peak flow readings appeared to be the main methods used to diagnose asthma, with more than half of the doctors surveyed reported conducting reversibility testing with peak flow metre. Only a minority had used spirometry, mainly due to lack of accessibility. Asthma action plans were still under-prescribed.

Primary care settings are ideally placed to identify and manage patients with asthma. We found four of the six clinics provided dedicated asthma clinics, run by the Family Medicine Specialists, doctors, nurses and pharmacists who were trained in asthma care. Although, the outcome of dedicated asthma services has yet to be explored in the Malaysian primary care, a Cochrane review including studies from high-income countries like the United Kingdom and Australia reported promising results^14^. Asthma management provided by dedicated asthma clinics was associated with reduced emergency visits^15^ and improved asthma control in Sweden and the United Kingdom^16^. Our study found only one clinic reported using a defaulter tracing system, despite evidences from earlier local studies in Malaysia and Korea that showed this system improved adherence on scheduled visits^17,18^, and reduced unscheduled visits for acute exacerbations^18^. Implementing a defaulter tracing system in our primary care setting is important to facilitate attendances of follow-up clinics as only one-third of patients with asthma attended routine follow-up care^2,19^. Resources, educational material, and equipment for asthma for example, asthma care and treatment protocols, asthma action plans, PEFR reference charts and patient education leaflets are important to assist and facilitate healthcare providers in the clinic when managing asthma. We found some of these, including asthma action plans, were not available in important clinic areas such as the emergency rooms, consultation rooms, and pharmacies. A previous local qualitative study found the main reasons for this were budget constraints and lack of prioritisation^20^. Another local study documented adequate numbers of peak flow metres and PEFR reference charts provided in the clinic, however, there was no spacer or placebo inhalers available for teaching patients^21^. Readily available asthma protocols and charts in specific clinic areas (e.g., consultation or emergency rooms) would help healthcare providers on consultation and treatment decisions.

Asthma diagnosis remains a challenge, because of its variability in nature^22^. The GINA guidelines recommend the use of spirometry as a tool to assess lung function^23^, but this assumes that spirometry is accessible, feasible and affordable in practice—which we found was not the case in any of our clinics. Studies conducted in Western countries found that accessibility was not the major issue, but rather underutilisation was due to lack of education and awareness of spirometer use and difficulties in interpreting the results^24–28^. Lack of confidence in use and interpretation was also reported a barrier in our study. One study reported the reason for underutilisation of spirometry testing was patient’s unwillingness to undergo the test due to time constraint^29^. A primary care study echoed our findings that a clinical history supported by peak flow rate assessment of variability was the main strategy to arrive at a diagnosis of probable asthma^30^. This could be a pragmatic approach to diagnosing asthma in primary care in limited resource settings but further evidence on the accuracy of this approach is needed.

There was inadequate availability of long-term controller medications specifically ICS/LABA inhalers in our health clinics, which limit the supply to only a few of the patients with poorly controlled asthma, and under the provision of asthma action plans—a challenge shared with other countries^31–33^. The healthcare providers, especially those involved in dedicated asthma services, had attended asthma training, carried out regular asthma audits and performed quality assurance programmes, however, the effectiveness and impact of these activities on practice to improve asthma care has yet to be explored. Existing studies have documented inconsistencies of clinic practices and skill-based trainings^21,34,35^.

This study highlights the gaps in resources, organisation, and practice, currently present in asthma care in our primary care setting. We suggest the following list of improvements in the delivery and organisation of asthma management:

i. **Increase accessibility to spirometry**. Correct asthma diagnosis is important for appropriate management; thus, it is important that spirometry and training in its use - is accessible in primary care settings. Currently spirometry is only provided at tertiary centres conducted by personnel certified by the Malaysian Thoracic Society^36^, so peak flow rate measurement is the main tool used to diagnose asthma in our low-resource primary care settings.
ii. **Use of peak expiratory flow rate (PEFR) and reversibility test**. In the absence of spirometry due to resource constraints, PEFR and reversibility test can be used as a pragmatic tool to diagnose asthma. Several studies have suggested serial PEFR over a 2–4-week period to assess the amplitude of airway variability more than 20% to support the diagnosis of asthma, with the important caveat that lack of PEFR variability does not rule out asthma (because of natural variability in asthma control)^6,23,37^. Interpretation should always be supported by typical history of probable asthma^38–40^. If clinical history and PEFR are used as diagnostic tool, suggestions on the diagnostic algorithm and continuous medical education are needed on technique, interpretation, validity and limitations of use to support implementation^40^. Despite being available for many years, more evidence is needed to support the practical use of PEFR reversibility testing and serial PEFR in diagnosing asthma.
iii. **Implement a defaulter tracing system**. To optimise patients’ adherence to follow-up asthma care, a defaulter tracing system should be in place to complement the existing appointment-based system. Several studies have shown regular asthma follow-up reduces the risk of exacerbation^17–19^. The use of telemedicine has a potential to improve follow-up care in asthma and asthma control^41^.
iv. **Develop an ‘asthma care kit’—a package to assist healthcare providers**. An asthma care kit that comprises of asthma education, assessment tools and treatment pathway protocols for both scheduled and unscheduled visits could potentially assist healthcare providers when reviewing patients. The ‘asthma care kit’ should be placed and easily reached by healthcare providers in all consultation and emergency rooms. There are promising results from implementation of an asthma care package in improving practice and patients outcomes^42,43^.
v. **Upskill healthcare providers on asthma diagnosis, treatment and communication skills, particularly on asthma self-management**. This can be done through regular audits, training and educational programmes. The evidence-based practice which includes asthma self-management^44,45^ needs to be encouraged, and long-term controller medications^23^ should be made accessible to enhance doctors’ practice in the treatment of poorly controlled asthma.

The strength of this study was the response rate of 94% was excellent. Nevertheless, several limitations were identified. Reasons behind the identified gaps in care were not explored; this includes an unavailable defaulter tracing system, inadequate provision of ICS/LABA in clinics, and variation in the number of resources, equipment and materials for asthma in the specified clinic rooms. This could be explored further qualitatively. In addition, only Family Medicine Specialists and the doctors were invited to participate in the survey because they were the most involved in asthma care at the time of data collection. The Family Medicine Specialists led and organised asthma care in the clinic. Diagnosing asthma, assessing asthma control, prescribing, and counselling were mainly carried out by the doctors.

It would have added value if other healthcare providers (e.g., nurses and pharmacists) completed the survey. In addition, it was a self-reported questionnaire; doctors’ actual practice in patients’ clinical records was not evaluated to observe the match between reported and actual practice.

This study has demonstrated opportunities to further improve the provision of healthcare resources, organisational support, educational materials, and equipment for asthma in our primary care setting. Spirometry was rarely utilised in asthma diagnosis as compared to the use of peak flow measurements with reversibility testing, suggesting that this is a practical alternative to spirometry in a low-resource setting. Reinforcing education on asthma action plan deserves to be addressed to ensure asthma care is optimised.

## Supplementary information

Reporting summary checklist

## Data Availability

The data that support the findings of this study are available from the corresponding author upon request.
